# Computerized Speechreading Training for Deaf Children: A Randomized Controlled Trial

**DOI:** 10.1044/2019_JSLHR-H-19-0073

**Published:** 2019-07-23

**Authors:** Hannah Pimperton, Fiona Kyle, Charles Hulme, Margaret Harris, Indie Beedie, Amelia Ralph-Lewis, Elizabeth Worster, Rachel Rees, Chris Donlan, Mairéad MacSweeney

**Affiliations:** a Institute of Cognitive Neuroscience, University College London, United Kingdom; b Deafness, Cognition and Language Research Centre, University College London, United Kingdom; c Division of Language and Communication Science, City University of London, United Kingdom; d Department of Education, University of Oxford, United Kingdom; e Faculty of Health and Life Sciences, Oxford Brookes University, United Kingdom; f Department of Language and Cognition, University College London, United Kingdom

## Abstract

**Purpose:**

We developed and evaluated in a randomized controlled trial a computerized speechreading training program to determine (a) whether it is possible to train speechreading in deaf children and (b) whether speechreading training results in improvements in phonological and reading skills. Previous studies indicate a relationship between speechreading and reading skill and further suggest this relationship may be mediated by improved phonological representations. This is important since many deaf children find learning to read to be very challenging.

**Method:**

Sixty-six deaf 5- to 7-year-olds were randomized into speechreading and maths training arms. Each training program was composed of a 10-min sessions a day, 4 days a week for 12 weeks. Children were assessed on a battery of language and literacy measures before training, immediately after training, and 3 months and 11 months after training.

**Results:**

We found no significant benefits for participants who completed the speechreading training, compared to those who completed the maths training, on the speechreading primary outcome measure. However, significantly greater gains were observed in the speechreading training group on one of the secondary measures of speechreading. There was also some evidence of beneficial effects of the speechreading training on phonological representations; however, these effects were weaker. No benefits were seen to word reading.

**Conclusions:**

Speechreading skill is trainable in deaf children. However, to support early reading, training may need to be longer or embedded in a broader literacy program. Nevertheless, a training tool that can improve speechreading is likely to be of great interest to professionals working with deaf children.

**Supplemental Material:**

https://doi.org/10.23641/asha.8856356

Speechreading refers to the ability to understand speech on the basis of visual, rather than auditory, perceptual information. More commonly known as lipreading, the term *speechreading* acknowledges the fact that there is more to understanding visual speech than solely what is seen on the lips (Arnold, 1997). For many profoundly deaf children, speechreading provides their main access to spoken language. For others, visual speech information can support speech perception by complementing impoverished auditory speech information provided via cochlear implants or digital hearing aids.

Given that speechreading provides visual access to spoken language, it is perhaps not surprising that speechreading has been argued to play an important role in deaf children's reading development (Kyle & Harris, 2006, 2010). Support for this comes from cross-sectional studies that have demonstrated concurrent correlations between speechreading and reading abilities in deaf children (Kyle, Campbell, & MacSweeney, 2016; Kyle & Harris, 2006) and adult readers of both English (Mohammed, Campbell, MacSweeney, Barry, & Coleman, 2006) and Spanish (Rodriguez-Ortiz, Saldaña, & Moreno-Perez, 2017). Furthermore, longitudinal studies have found predictive relationships between early speechreading skills and later reading outcomes in young deaf children (Harris, Terlektsi, & Kyle, 2017; Kyle & Harris, 2010, 2011); better speechreading skills are associated with better subsequent reading outcomes. Data from the Kyle and Harris (2010) longitudinal study further suggested that this relationship between speechreading and reading is mediated by phonological processing in deaf children. Information about the sublexical structure of speech derived from speechreading may contribute to the formation of phonological representations of spoken language in deaf children, which they can then bring to the task of learning to read (see Kyle, 2015). This is important since the vast majority of deaf children find learning to read to be a difficult task, with many studies reporting significantly poorer reading skills in deaf children than their hearing peers (Qi & Mitchell, 2011; Wauters, Van Bon, & Tellings, 2006). This raises the possibility that training and improving speechreading skills in young deaf children could support the development of their phonological and reading skills.

However, whether it is even possible to train speechreading skills has been the focus of debate following discrepant findings. There are very few studies of speechreading training with deaf adults. Where speechreading gains have been reported following speechreading training, in contrast to a control group, these have often been small (e.g., Bernstein, Auer, & Tucker, 2001). Even less published evidence is available regarding speechreading training in deaf children, despite researchers highlighting its potential benefits (Arnold, 1993). Van Uden (1983) trained profoundly deaf 8- to 14-year-olds on a mixed speechreading/articulation program. Participants who viewed themselves producing speech made greater speechreading gains than groups that viewed a teacher producing speech.

Despite the paucity of high-quality evidence from speechreading training studies, numerous studies have demonstrated a speechreading advantage for adults who have experienced congenital or early-onset deafness compared to typically hearing adults (Auer & Bernstein, 2007; Bernstein, Demorest, & Tucker, 2000; Mohammed et al., 2006; Pimperton, Ralph-Lewis, & MacSweeney, 2017). This suggests that increased experience of and attention to visual speech early on in life can result in a form of perceptual compensation and bring about improvements to visual-only speech perception. This is consistent with studies in other modalities, which have indicated enhanced perceptual compensation at earlier ages (e.g., Gougoux et al., 2004). The responsivity of speechreading skill to early environmental experience supports the possibility that it may be amenable to training in young children.

In the current study, we created and evaluated, in a randomized controlled trial (RCT), a 12-week computer-based adaptive speechreading training program for 5- to 7-year-old deaf children. In order to maximize the chances of the speechreading training supporting the development of phonological and reading skills, part of the training included explicit linking of visual speech to phonemes and graphemes. This study allowed us to test two key hypotheses:

1. Speechreading skills in young deaf children can be improved by training.
2. Speechreading training can lead to improvements in phonological and reading skills.

## Method

### Design

A single-blind RCT was conducted with deaf children aged 5–7 years. Children were tested on an assessment battery prior to training (T1) and then randomized to complete either speechreading (silent speech) training or maths training (active control). Follow-up assessments were conducted immediately following the completion of the intervention (T2) and 3 months later (T3). The study design, analysis plan, and sample size calculations were preregistered on the Open Science Framework (https://osf.io/ygz7f/). Following data collection at T3, it was decided to test the children again 11 months after the completion of the intervention (T4) to examine the durability of any intervention effects. Although not registered in the Open Science Framework study design, these data are also reported here for completeness. Ethical approval for the study was provided by the University College London Research Ethics Committee. Informed consent was obtained from all parents/caregivers of the children involved in the study. Children also gave their assent to be involved and were informed they were able to stop their involvement at any point.

### Participants

Sample size was determined based on a formal power calculation that showed that, with *N* = 28 in each arm, there was a better than 80% power to detect a difference between groups equivalent to *d* = 0.5 (*p* < .05, two-tailed). This sample size was increased to 32 per group to allow for an anticipated dropout rate of 10%.

Schools for deaf children, mainstream schools with resource bases (hearing impaired units) for deaf children, and local authority support services for deaf children were asked to identify children who met the following eligibility criteria:

1. aged between 5 and 7 years at the time of the first assessment,
2. with a severe or profound bilateral hearing loss that had onset before the age of 12 months, and
3. able to meet the physical and attentional demands of playing a computer game for 10 min a day.

**Figure 1.**
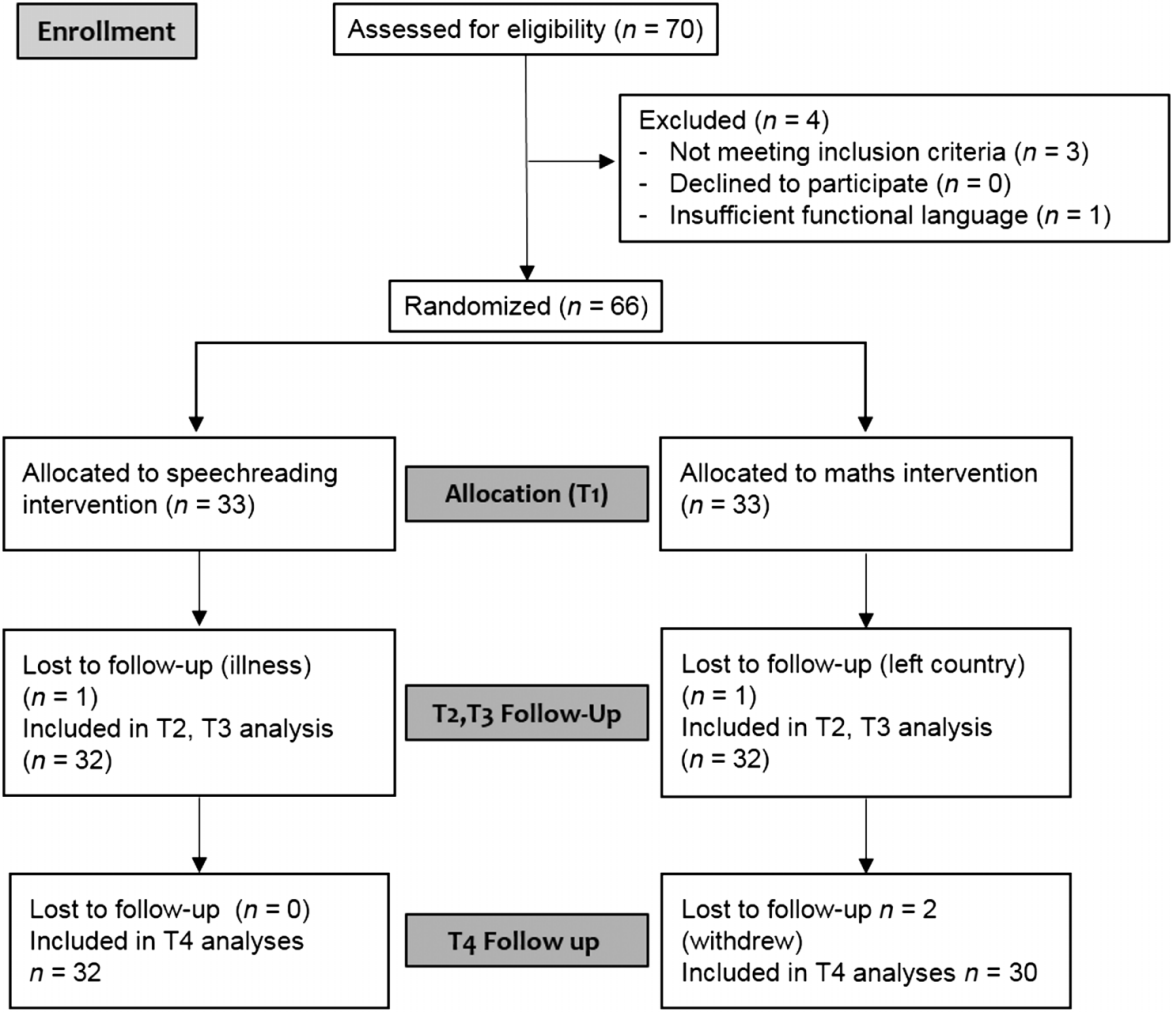
Flowchart documenting movement of participants through the phases of the trial.

The caregivers of 70 deaf children provided informed consent for their child to participate in the trial. The CONSORT diagram in Figure 1 shows the flow of participants through the trial. See Supplemental Material S1 for the CONSORT checklist.

### Randomization and Blinding

Four participants were excluded prior to randomization. One had insufficient functional language skills to complete any assessments, two did not meet the audiological inclusion criteria, and one did not meet the age inclusion criteria. The remaining 66 participants were randomized into the two arms of the trial (intervention = 33, control = 33). Group allocation was conducted independently by the University of York Trials Unit, using minimization (1:1 allocation ratio) on the following measures:

1. Test of Child Speechreading (ToCS; Kyle, Campbell, Mohammed, Coleman, & MacSweeney, 2013) total score at T1 (above vs. below a median split),
2. communication preference (oral vs. sign or speech with sign; determined for each child on the basis of a language use questionnaire completed by teachers), and
3. year group (< Year 2 vs. ≥ Year 2; in the United Kingdom, Year 2 is the academic year that the child turns 7 years old).

The researchers carrying out the assessments of the participants on the study outcomes were blind to the group allocation of the participants.

### Baseline Characteristics and Retention

Demographic, audiological, and educational characteristics of the intervention and control groups are shown in Table 1. Two children (one intervention, one control) were lost to follow-up after T1 (see Figure 1) and did not provide assessment data at T2 and T3. A further two children, both in the active control group, were lost to follow-up at T4.

**Table 1.** Participant characteristics of the intervention and control groups at baseline (Time 1).

Characteristic	Intervention (*n* = 33)	Active control (*n* = 33)
Chronological age (months), *M* (*SD*), range	73.24 (8.08), 61–94	71.94 (7.68), 59–91
Nonverbal ability (raw score), *M* (*SD*), range	6.24 (2.29), 2–14	6.82 (2.63), 1–14
Year group (%)		
< Year 2^a^	55	58
≥ Year 2	45	42
Communication preference (%)		
Spoken English only	30	30
Sign or sign with speech	70	70
School setting (%)		
School for deaf children	18	21
Resource base (HIU)	61	58
Mainstream school	21	21
Device use (%)		
No device	6	6
Bilateral CIs	48	39
One HA, one CI	6	0
Bilateral HAs	39	55
Unaided category of deafness (%)		
Severe^b^	42	33
Profound	58	67

*Note.* HIU = hearing impaired unit; CI = cochlear implant; HA(s) = hearing aid(s).

a Year 2: In the United Kingdom, Year 2 is the academic year that children turn 7 years old.

b Five children (two in the speechreading group, three in the maths group) had a hearing loss that at their latest hearing assessment was in the moderate category in their better ear but severe or profound in the contralateral ear; these children were included in the severe category.

### Interventions

**Figure 2.**
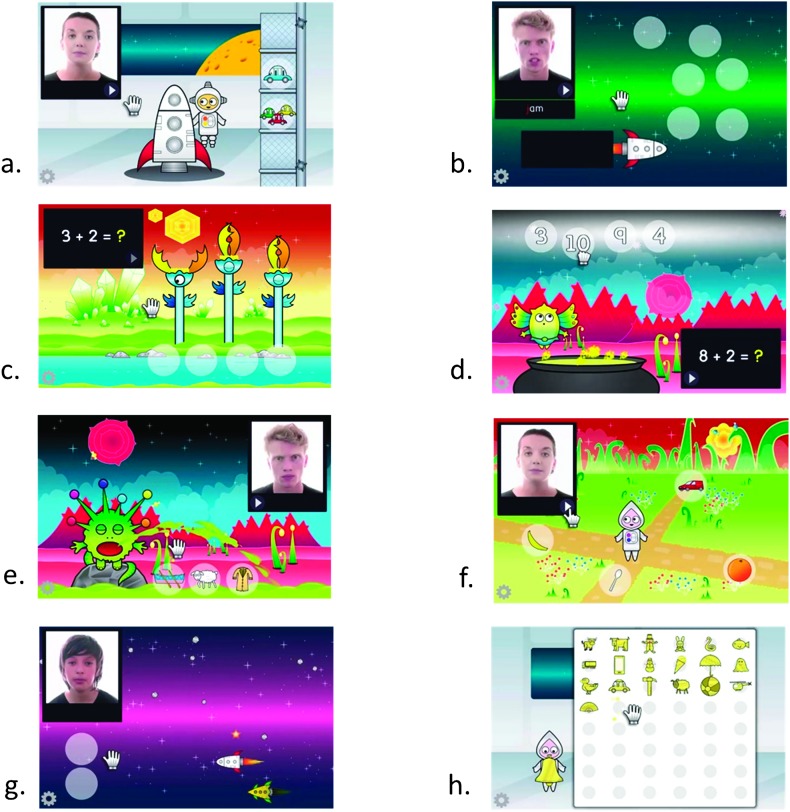
Screenshots from each of the seven computer games (a–g) that were used in the intervention and control conditions of the trial and from one of the reward scenes (h). Example content from both the speechreading and number and maths interventions is shown in the seven games. Note that stimulus (speech video or math problem) and targets (pictures, letters, or numbers) did not typically appear on the screen at the same time (except in Figure 2d), hence the appearance of the screenshots.

Both the speechreading and maths interventions were run within a suite of seven space-themed computer games (see Figure 2 for examples and Supplemental Material S1 for full details). Adaptive algorithms were established to enable a child to progress through the program of training sessions at a pace appropriate to their ability. The games were designed to run across forty-eight 10-min sessions. Each 10-min session was packaged so that there was a narrative structure to the space games and the child received a virtual reward at the end of each session to collect in an online “trophy cabinet.”

In the speechreading training, the first 16 sessions contained trials that involved visual speech videos and pictures only. These focused on introducing the vocabulary used in the intervention (103 words) and on mapping speechread words to a corresponding image (video-to-picture matching). A subset of trials also involved picture-to-video (speechread word) matching. For all trials, participants were free to articulate the perceived words if they chose to do so. An algorithm was developed that enabled the difficulty level of these trials to be systematically varied in an adaptive way based on the child's performance. In addition to trials operating at the single word level, children also completed trials that (a) showed videos of two-word utterances (e.g., “red hat,” “blue door”) and (b) showed videos of the two-word utterances within a carrier sentence and hence required the child to identify the key information in the surrounding sentence (e.g., “find the red hat this time”).

Sessions 17–48 continued the speechreading training trials introduced in the first 16 sessions but additionally included trials that contained orthographic stimuli and that focused on training mappings between visual speech patterns and letters and words. Full details about both interventions can be found in Supplemental Material S2.

#### In-Game Assessments

Within both the speechreading and maths intervention programs, there were built-in assessments of speechreading skills every eight training sessions to enable “online” tracking of changes in speechreading performance while the intervention was ongoing (i.e., between the T1 and T2 assessment time points). In each assessment trial, the children viewed a video of either a trained or an untrained talker saying one of the trained words and had to choose the corresponding picture from a choice of four. 

### Primary Outcome Measure: Speechreading

The prespecified primary outcome measure in this study was children's standard score on the ToCS (Kyle et al., 2013). This was selected as the primary outcome measure to test the primary hypothesis of the study: that speechreading skills in young deaf children can be improved by training. The ToCS is a standardized computerized assessment of speechreading ability that comprises three subtests: single words, sentences, and short stories. In all tasks, the participant is required to match a silent video clip of a spoken target to a picture. At T4, children only completed one of these subtests, the ToCS single-word subtest. More details of each assessment are provided in Supplemental Material S2.

### Secondary Outcome Measures

The following prespecified secondary outcome measures were also collected to provide additional information about the impact of the training on children's speechreading, language, reading, and mathematical skills. Additional details for each measure are provided in Supplemental Material S2.

#### Speechreading: ToCS—Everyday Questions Test

The children completed the Everyday Questions subtest from the ToCS. This required them to watch silent videos (*n* = 12) of two talkers saying questions they might encounter in everyday life (e.g., Where do you live?) and tell the experimenter what they thought the question was. Children received two scores on this measure, one reflecting the number of questions on which they correctly reproduced the gist of the sentence (ToCS Everyday Questions Items Correct Gist) and one reflecting the total number of individual words that the child got correct across all 12 questions out of a possible 62 (ToCS Everyday Questions Words Identified).

#### Vocabulary

A naming task, using the pictures from the training (*N* = 74), was used to assess participants' knowledge of the vocabulary used in the speechreading training. Children could respond in their preferred language. If they named it in sign, they were asked if they knew the English word. Each participant was given a score for the number of correct items produced in spoken English (Spoken Vocabulary; total = 74) and a score for the number of correct items produced either in spoken English or British Sign Language, thus providing a measure of overall vocabulary, regardless of modality (Overall Vocabulary; total = 74).

#### Audiovisual Speech Production

Participants were filmed completing the picture-naming task used to assess vocabulary. These videos were used to establish a measure of the quality of the child's audiovisual (AV) speech production derived from 30 of the trained words that were selected to maximize the range of phonemes in syllable-initial and syllable-final positions and to provide a range of word lengths and syllable structures, including consonant clusters. For each child, a percentage score was calculated that reflected changes in the quality of their speech production of the same words over time. This measure provided an indirect measure of the quality of the child's phonological representations (Stackhouse & Wells, 1997).

#### Phonological Awareness

A novel phonological awareness task, based on that of Kyle and Harris (2006), was developed using stimuli from the speechreading training to assess children's awareness of spoken English phonology at the level of onset and rime.

#### Letter–Sound Knowledge

The Letter–Sound Knowledge subtest of the York Assessment of Reading for Comprehension–Primary School Edition (Snowling et al., 2009) was used to assess children's knowledge of the correspondence between letters and sounds. This assessment was not administered at T4.

#### Word Reading

The Early Word Recognition Test and the Single Word Reading Test from the York Assessment of Reading for Comprehension (Snowling et al., 2009) were used. In addition, we developed a test to assess single word reading for stimuli included in the speechreading training (*n* = 24 trials). Children saw a word in the middle of the screen and had to point to the corresponding picture from a choice of four; therefore, no speech production was required. A reading composite score was created by summing each child's *z* scores on the three word reading measures.

#### Number Skills

Three measures of number skills were administered: (a) the Early Number Concepts section of the British Ability Scales–Third Edition (Elliot & Smith, 2011), (b) the Test of Basic Arithmetic and Numeracy Skills (Hulme, Brigstocke, & Moll, 2016), and (c) children were asked to count to 30, with the highest number they could reach being their score on this task. A Number Skills composite score was created by summing each child's *z* scores on the three measures of number skills. At T4, only the measure of addition and subtraction fluency was administered.

### Statistical Methods

Differences between the intervention and control groups on the outcome variables at T2, T3, and T4 were tested using analyses of covariance (ANCOVAs). The outcome variable at T2, T3, or T4 was the dependent variable, performance on the same variable at baseline (i.e., T1) and the three randomization stratifiers (ToCS score, communication preference, school year group) were covariates, and group (i.e., intervention vs. control) was a fixed factor. All ANCOVA models were run with bootstrapped standard errors (200 bootstrap samples). Equality of slopes for the models was assessed by including the interaction between covariate and group. Cohen's *d* provided a measure of the intervention effect size and was calculated by dividing the difference in progress between the intervention and control groups by the pooled initial standard deviation (Morris, 2008).

## Results

Assessment data were collected from 32 children in each group at T1, T2, and T3. At T4, data were collected from 32 children in the speechreading group and 30 children in the control group.

The mean time between the first two assessment points (T1 and T2) was 5.42 months (*SD* = 1.21). The wide range within the whole sample (3–8 months) was a result of school logistical constraints and child illness. However, there were no significant differences between the intervention and control groups in terms of their T1–T2 distance (speechreading: *M* = 5.34, *SD* = 1.18, range = 3–7; maths: *M* = 5.50, *SD* = 1.24, range = 3–8), *t*(62) = 0.52, *p* = .61, bootstrapped bias-corrected and accelerated (BCA) 95% CI [−0.76, 0.42]. The distance between T2 and T3 in the whole sample was less variable (*M* = 2.66, *SD* = 0.48, range = 2–3), and there were no significant differences between the intervention and control groups (speechreading: *M* = 2.69, *SD* = 0.47, range = 2–3; maths: *M* = 2.63, *SD* = 0.49, range = 2–3), *t*(62) = 0.52, *p* = .61, bootstrapped BCA 95% CI [−0.17, 0.31]. The distance between the T3 and T4 assessments averaged 7.92 months (*SD* = 0.91, range = 6–10) and did not differ significantly between the intervention (*M* = 7.94, *SD* = 0.98, range = 6–10) and control (*M* = 7.90, *SD* = 0.84, range = 6–10) participants, *t*(60) = 0.16, *p* = .87, bootstrapped BCA 95% CI [−0.43, 0.48].

### Adherence to Intervention

There was substantial variation in the number of training sessions completed by children in the speechreading training group (*M* = 36.77, *SD* = 16.88, range = 0–48). This was primarily due to school logistical and technological constraints and child illness. There were no significant differences between the two groups in total number of intervention sessions completed (speechreading: *M* = 35.25, *SD* = 18.61; maths: *M* = 38.28, *SD* = 15.11), *t*(62) = 0.72, *p* = .48, *d* = 0.18. Six children did not complete any sessions due to school technology issues but were still included in the intention-to-treat analyses. Very similar numbers of children completed all 48 training sessions in the speechreading and maths training groups (speechreading = 18; maths = 19).

#### In-Game Assessments

Table 2 shows the mean performance on the seven in-game speechreading assessments (all trials, trained talker trials, and untrained talker trials) for the 37 children (speechreading = 18, maths = 19) who completed all of the training sessions and therefore had a complete set of in-game assessment (IGA) data. Cohen's *d* effect sizes examining the difference in progress between groups from the first IGA (completed prior to the first training session) to the final IGA (completed after the final training session) indicated a small effect in favor of the speechreading training group (*d* = 0.45) on all trials combined. Looking separately at the trained versus untrained talker trials, there was a large beneficial effect of speechreading training on trained talker trials (*d* = 0.80) but no effect on the untrained talker trials (*d* = 0.17) immediately following the training.

**Table 2.** Means and standard deviations for performance on the in-game assessments (*n* = 7) for the children who participated in all of the training sessions.

IGA	Stimulus words	Intervention (*n* = 18)		Active control (*n* = 19)	
		*M*	*SD*	*M*	*SD*
IGA1	Total	12.83	3.87	11.58	3.79
	Trained	6.83	1.76	6.47	2.61
	Untrained	6.00	2.89	5.11	1.59
IGA2	Total	13.83	5.20	11.63	4.22
	Trained	7.17	3.03	6.00	3.04
	Untrained	6.67	2.72	5.63	1.86
IGA3	Total	14.11	3.76	13.16	4.41
	Trained	7.56	2.28	6.79	2.78
	Untrained	6.56	1.89	6.37	2.24
IGA4	Total	14.33	5.03	13.26	4.62
	Trained	7.44	2.83	6.95	2.55
	Untrained	6.89	2.49	6.32	2.81
IGA5	Total	14.83	4.31	12.68	5.02
	Trained	7.67	2.70	6.47	2.89
	Untrained	7.17	2.33	6.21	2.44
IGA6	Total	14.94	5.62	11.95	3.95
	Trained	8.11	3.10	6.00	2.43
	Untrained	6.83	2.98	5.95	2.50
IGA7	Total	14.72	4.69	11.74	4.19
	Trained	7.39	2.68	5.68	2.16
	Untrained	7.33	2.40	6.05	2.57

*Note.* Data are provided for overall performance and for the trained and untrained talkers separately. Total Max = 30; Trained/Untrained Max = 15. IGA = in-game assessment.

### Group Comparisons on Outcome Measures—Intention-to-Treat Analyses

Descriptive statistics, including means and standard deviations, for the performance of all participants on the outcome measures at baseline (T1), immediate follow-up (T2), and delayed follow-up (T3) are presented in Table 3; and for the exploratory second delayed follow-up (T4), in Table 4. Also presented are Cohen's *d* effect sizes and results of the ANCOVAs comparing the two groups on each outcome measure while adjusting for their baseline performance and the three randomization stratifiers.

**Table 3.** Intention-to-treat analyses: means and standard deviations, for all participants on the outcome measures at baseline (T1), immediate follow-up (T2), and delayed follow-up (T3).

Outcome measure	Intervention (*n* = 32)		Active control (*n* = 32)		Cohen's *d* ^a^	β	*p*	95% CI
	*M*	*SD*	*M*	*SD*				
ToCS total standard score (*M* = 100, *SD* = 15)								
T1	94.78	10.96	95.84	13.48			.81	[−4.12, 5.27]
T2	93.53	11.14	94.13	12.48	0.04	0.57	.67	[−3.66, 5.71]
T3	95.16	11.88	95.03	12.79	0.10	1.03		
ToCS Everyday Questions								
*Words identified (max = 62)*								
T1	5.50	9.27	5.53	11.13				
T2	10.41	10.58	8.97	12.79	0.14	1.83	.40	[−2.46, 6.13]
T3	14.16	12.40	10.34	14.70	0.38	4.38	.07	[−0.42, 9.18]
*Items correct gist (max = 12)*								
T1	0.41	0.95	0.75	1.98				
T2	1.28	1.73	1.06	2.03	0.38	0.44	.18	[−0.21, 1.09]
T3	1.78	2.28	1.22	2.28	0.61	0.86	.03	[0.10, 1.61]
Vocabulary (max = 74)								
*Overall*								
T1	54.50	5.78	53.78	9.33				
T2	63.66	6.56	60.69	8.76	0.30	2.69	.02	[0.48, 4.89]
T3	63.59	7.31	62.81	8.26	0.008	0.67	.60	[−1.82, 3.16]
*Spoken*								
T1	41.78	17.15	37.22	22.33				
T2	53.00	20.38	44.63	23.70	0.19	3.40	.04	[0.09, 6.71]
T3	54.69	18.84	49.16	22.59	0.05	0.85	.64	[−2.75, 4.46]
AV speech production^b^ (%)								
T1	65.22	31.67	56.23	31.23				
T2	69.80	28.65	58.94	33.24	0.06			
T3	72.14	30.20	60.15	33.67	0.10	See note^c^		
Phonological awareness (max = 24)								
T1	10.28	4.23	10.41	5.28				
T2	12.88	5.53	12.16	6.07	0.18	0.95	.28	[−0.79, 2.70]
T3	14.31	5.83	13.03	5.92	0.30	1.54	.09	[−0.25, 3.34]
Letter–sound knowledge (max = 17)								
T1	11.34	4.99	10.47	5.70				
T2	13.03	5.14	12.03	6.02	0.02	0.15	.78	[−0.89, 1.18]
T3	13.31	5.53	11.81	6.20	0.12	0.67	.31	[−0.63, 1.96]
Word Reading (*z*-score composite)								
T1	0.01	2.89	−0.01	2.90				
T2	0.12	2.73	−0.12	3.02	0.08	0.26	.34	[−0.27, 0.80]
T3	0.12	2.71	−0.12	2.99	0.08	0.28	.36	[−0.32, 0.87]
Number Skills (*z*-score composite)								
T1	0.08	2.54	−0.08	2.54				
T2	0.02	2.51	−0.02	2.58	−0.06	−0.08	.81	[−0.73, 0.57]
T3	0.001	2.35	−0.001	2.66	−0.06	−0.10	.77	[−0.78, 0.58]

*Note.* Also presented are Cohen's *d* effect sizes and results of the analyses of covariance comparing the two groups on each outcome, at T2 and T3, while adjusting for their baseline performance. CI = confidence interval; ToCS = Test of Child Speechreading; T1 = Time 1; T2 = Time 2; T3 = Time 3; AV = audiovisual.

a Cohen's *d*: Difference in progress between groups divided by pooled initial standard deviation.

b *n* = 30 in each group.

c Analyses of covariance did not meet equal slopes assumption. See Results for further details.

**Table 4.** Intention-to-treat analyses: means and standard deviations, for participants on the outcome measures at baseline (T1) and the follow-up, 11 months after training (T4).

Outcome measure	Intervention (*n* = 32)		Active control (*n* = 30)		Cohen's *d* ^a^	β	*p*	95% CI
	*M*	*SD*	*M*	*SD*				
ToCS Single Words subtest (max = 15)								
T1	7.28	2.49	7.60	2.61				
T4	8.75	2.84	8.70	2.87	0.14	0.29	.63	[−0.89, 1.46]
ToCS Everyday Questions								
*Words identified (max = 62)*								
T1	5.50	9.27	5.90	11.41				
T4	18.13	12.38	15.37	17.96	0.31	3.46	.22	[−2.02, 8.94]
*Items correct gist (max = 12)*								
T1	0.41	0.95	0.80	2.04				
T4	2.41	2.27	2.47	3.10	0.22	0.38	.43	[−0.56, 1.32]
Vocabulary (max = 74)								
*Overall*								
T1	54.50	5.78	53.57	9.47				
T4	63.34	6.11	63.33	7.51	−0.12	−0.29	.83	[−2.84, 2.27]
*Spoken*								
T1	41.78	17.15	37.60	21.50				
T4	55.47	18.55	52.60	19.21	−0.08	−1.13	.55	[−4.80, 2.54]
AV speech production^b^ (%)								
T1	65.67	31.97	59.74	30.48				
T4	74.57	32.95	68.10	31.91	0.02	0.72	.86	[−7.34, 8.77]
Phonological awareness (max = 24)								
T1	10.28	4.23	10.77	5.19				
T4	14.59	5.36	14.30	5.57	0.17	0.73	.40	[−0.96, 2.42]
Word Reading (*z*-score composite)								
T1	0.01	2.89	0.05	2.95				
T4	0.29	2.58	−0.31	3.05	0.22	0.74	.08	[−0.08, 1.56]
Arithmetic fluency (max = 60)								
T1	4.25	4.71	4.77	6.07				
T4	13.19	10.17	14.00	11.95	−0.05	−0.31	.83	[−3.13, 2.50]

*Note.* Also presented are Cohen's *d* effect sizes and results of the analyses of covariance comparing the two groups on each outcome at T4 while adjusting for their baseline performance. CI = confidence interval; ToCS = Test of Child Speechreading; T1 =Time 1; T4 = Time 4; AV = audiovisual.

a Cohen's *d*: Difference in progress between groups divided by pooled initial standard deviation.

b *n* = 30 in each group.

There were no significant differences between the intervention and control groups on the prespecified primary outcome variable, total ToCS standard score, at either T2 (*d* = 0.04) or T3 (*d* = 0.10) nor on the ToCS Word subtest at T4 (*d* = 0.14). Of the prespecified secondary outcome variables, there was evidence of significant benefit of speechreading training on the ToCS Everyday Questions task at T3 (*d*_Items_ = 0.61, *d*_Words_ = 0.38; see Table 2). Effects were smaller at T4, 11 months after training ended (*d*_Items_ = 0.22, *d*_Words_ = 0.31; see Table 3), and no longer significant.

There were significant beneficial effects of speechreading training on overall vocabulary (*d* = 0.30) and spoken vocabulary (*d* = 0.19) at T2 (see Table 2), but no evidence of sustained effects at T3 (*d* < 0.01 and *d* = 0.05, respectively) or T4 (*d* = −0.12 and *d* = −0.08, respectively). The effects of training on phonological awareness and word reading were all small and nonsignificant (*d*s = 0.08–0.30). Finally, there was no evidence of an effect of intervention group on Letter–Sound Knowledge (T2/T3) or Number Skills (T2/T3/T4) (*d*s = −0.06 to 0.12).

The ANCOVA model for AV speech production at T2 did not meet the necessary assumption for ANCOVA of equal slopes (i.e., no interaction between the covariate and the dependent variable). The interaction between intervention group and the covariate (T1 AV speech production) was significant (unstandardized slope = 0.17, 95% CI [0.05, .29], *p* = .005), indicating a shallower slope in the intervention group than the maths control group. This pattern indicated that the speechreading intervention was more effective for children starting with lower scores on this measure. Follow-up tests showed that the groups did not differ at posttest at the mean of the covariate, *F*(1, 53) = 1.53, *p* = .22. However, for children scoring at 1 *SD* below the mean of the covariate, there was a significant advantage for the intervention group, *F*(1, 53) = 8.55, *p* = .005. This pattern needs to be interpreted with caution but suggests that speechreading training improved T2 AV speech production in children who started with particularly low scores on this measure. A similar pattern was seen for the same variable at T3, with a significant advantage to the intervention group for children scoring at 1 *SD* below the mean of the covariate, *F*(1, 56) = 4.88, *p* = .03, and no significant group differences at the mean of the covariate, *F*(1, 56) = 1.73, *p* = .19, though the interaction term between intervention group and the covariate was not significant (unstandardized slope = 0.14, 95% CI [−0.02, 0.30], *p* = .08).

### Group Comparisons on Outcome Measures—Completing Participants Only

As already described, intervention compliance was found to be variable. This is likely to have reduced the effectiveness of the intervention. A similar number of children fully completed each type of training: speechreading, *n* = 18; maths, *n* = 19. Therefore, we carried out additional exploratory analyses to examine whether effect sizes were larger in the subset of participants who completed the full intervention (see Table 5).

**Table 5.** Comparison of participants who completed all training sessions: intervention (*n* = 18) and active control (*n* = 19).

Outcome measure	Intervention (*n* = 18)		Active control (*n* = 19)		Cohen's *d* ^a^
	*M*	*SD*	*M*	*SD*	
ToCS total standard score					
T1	94.11	9.46	95.89	12.89	
T2	92.83	10.37	93.95	10.88	0.06
T3	93.83	10.55	96.16	12.40	−0.05
ToCS Everyday Questions					
*Words identified (max = 62)*					
T1	6.78	10.33	4.16	6.53	
T2	11.44	10.68	7.16	10.69	0.19
T3	17.39	12.23	8.05	12.27	0.78
T4	20.44	11.86	13.05	15.24	0.55
*Items correct gist (max = 12)*					
T1	0.50	1.15	0.37	0.83	
T2	1.28	1.53	0.47	1.02	0.68
T3	2.17	2.38	0.74	1.59	1.29
T4	2.72	2.42	2.00	2.29	0.59
Vocabulary (max = 74)					
*Overall*					
T1	54.89	5.73	56.16	6.69	
T2	65.83	5.57	64.21	4.43	0.46
T3	64.89	7.76	65.47	5.67	0.11
T4	63.94	6.39	65.79	4.09	−0.09
*Spoken*					
T1	45.50	15.53	39.00	21.16	
T2	58.78	18.10	45.58	23.82	0.36
T3	59.00	18.53	50.16	22.56	0.13
T4	59.61	16.66	53.58	19.78	−0.03
AV speech production^b^ (%)					
T1	68.62	30.69	53.06	29.86	
T2	74.56	26.68	55.16	32.72	0.13
T3	78.83	27.03	55.05	33.09	0.27
T4	80.88	27.28	59.47	33.44	0.19
Phonological awareness (max = 24)					
T1	10.78	4.14	10.53	4.68	
T2	13.83	5.59	12.47	5.17	0.25
T3	15.11	6.11	13.37	5.71	0.34
T4	15.78	5.71	14.47	5.23	0.24
Letter–sound knowledge (max = 17)					
T1	12.06	4.39	11.05	5.79	
T2	13.83	4.18	12.84	5.90	−0.004
T3	14.17	4.81	12.58	6.15	0.11
Word Reading (*z*-score composite)					
T1	0.55	3.33	0.12	2.32	
T2	0.76	2.94	0.36	2.52	−0.01
T3	0.86	2.86	0.46	2.49	−0.01
T4	0.80	2.67	0.41	2.28	−0.01
Number Skills (*z*-score composite)					
T1	0.19	2.28	0.22	1.98	
T2	0.45	2.09	0.27	1.98	0.10
T3	0.35	2.00	0.43	1.96	−0.02

*Note.* ToCS = Test of Child Speechreading; T1 = Time 1; T2 = Time 2; T3 = Time 3; AV = audiovisual.

a Cohen's *d*: Difference in progress between groups divided by pooled initial standard deviation.

b *n* = 16 speechreading, *n* = 17 maths.

For the variables where significant effects of the speechreading intervention were found in the intention-to-treat analyses involving all participants (ToCS Everyday Questions at T3; Vocabulary at T2), larger effect sizes were seen for the group comparisons within the subset of participants who completed the intervention. There were large effects of speechreading training on the ToCS Everyday Questions task at T3 (*d*_Items_ = 1.29, *d*_Words_ = 0.78); these were still apparent as medium-sized effects in favor of the speechreading trained group at T4 (*d*_Items_ = 0.59, *d*_Words_ = 0.55). The effects of speechreading training on T2 vocabulary were also larger in those participants who completed the intervention (*d*_overall_ = 0.46, *d*_spoken_ = 0.36) than when all children were included in the intention-to-treat analyses. The overall pattern of effects paralleled those analyses in not persisting beyond the T2 immediate follow-up assessment time point.

## Discussion

We examined the efficacy of a 12-week computerized speechreading training intervention for deaf children using an RCT. Our first hypothesis was that we would see gains in speechreading skills following the speechreading intervention. There was no evidence of effects of the intervention on our prespecified primary outcome variable, standard score on the ToCS, at any time point postintervention. However, the speechreading intervention group did show significant gains relative to the control group on the Everyday Questions Speechreading Test (a prespecified secondary outcome variable that involved speechreading untrained talkers and untrained items) 3 months after the end of training. The effects of speechreading training on this measure were smaller and no longer significant 11 months after training. At both time points, the intervention effects were larger in exploratory analyses, which included only those participants who had completed all the training sessions.

To our knowledge, this is the first RCT to evaluate a speechreading intervention with young deaf children. Significant effects of training on a speechreading measure that involved untrained models and untrained stimuli (Everyday Questions Test) in our intention-to-treat analyses suggest transfer of the training effects beyond the items and models that were included in the intervention. These data provide initial support for the efficacy of this computerized speechreading training program in boosting speechreading skills in young deaf children. The finding of a significant advantage to the speechreading intervention group on the secondary outcome Everyday Questions Speechreading measure, but no effects of the intervention on the primary outcome core ToCS, does however mean that confirmation of efficacy in a subsequent RCT would be of value. The discrepant findings may be explained by differences in response format between the two measures. The ToCS involved a forced-choice, closed-set response format in which children could guess the answer. By contrast, the Everyday Questions measure scored a free response and as such may offer a more valid measure. However, the Everyday Questions task was difficult for many of the children, which may have limited its sensitivity to detect changes in speechreading skill for the lower performers. Future studies of speechreading with children of this age may therefore benefit from additionally using a single-word, free response speechreading task.

An important question concerns the mechanism underpinning the improvements to speechreading skills associated with speechreading training. One possibility is that the training teaches children to attend better to speaking faces and that doing so enables them to perform better at extracting visual speech information from the talker. General improvements in visual attention were controlled for by the fact that the same gaming environment was used in both the speechreading and maths (control) training conditions. However, there were various features of the speechreading training that were designed to encourage the children to attend specifically to the face (see Supplemental Material S2). Consistent with this, eye-tracking data from children who participated in the current study that are reported elsewhere indicated that, at the T4 testing session (11 months after the end of training), children in the speechreading training group looked at the face significantly more than children in the control group (Worster et al., 2018). However, a lack of baseline eye-tracking data, before training, means that it is not possible to confirm that the training drove this group difference.

As well as improving attention to moving faces, it is possible that the speechreading training also led to improvements in working memory, which was necessary to achieve success on the training games. Previous studies with hearing children and deaf children have a shown positive relationship between speechreading skill and working memory (Heikkilä, Lonka, Ahola, Meronen, & Tiippana, 2017; Tye-Murray, Hale, Spehar, Myerson, & Sommers, 2014). Therefore, improvements in working memory via the training games may have further supported speechreading gains. Memory measures were not included in the battery of tests used in the current study due to time limitations. Future training studies should include such measures in order to further understand the possible role of working memory skills in gains following speechreading training.

Another and a likely complementary mechanism that may underlie the speechreading improvements is that it directly improved the ability of children to discriminate visually presented phonemes and thereby access more well-specified information from visual speech. This is likely since the training was specifically designed to improve these skills. In support of this mechanism, in children with the poorest speech output at the start of the training, speechreading training led to improvements in their AV speech output and therefore, by inference, in their phonological representations.

Performance on the in-game speechreading assessments indicated that, by the end of the intervention, children who completed the speechreading training showed advantages relative to the children who completed the maths training when speechreading trained, but not untrained, talkers. Yet, at 3 months posttraining, effects of speechreading were shown to have generalized to unknown speakers. Since all the models by necessity produce speech differently, visemic contrasts may initially be learned as speaker specific but then take time to generalize to other speakers. We had little control over the conditions under which the data from the IGAs were collected, and thus, these findings should be treated with caution. Nevertheless, taken together, these data suggest that transfer effects to speechreading of unfamiliar people, either via increased attention to faces, improvements in working memory, or increased discrimination of visual speech, may take time following training to fully manifest.

In summary, the data suggest that it is possible to improve speechreading skills in young deaf children using a computerized online training program. Although the current study cannot definitively confirm what precise mechanism or mechanisms led to the improvement in speechreading seen here after training, it does allow the generation of specific hypotheses for future testing. Further studies are needed to confirm these promising early findings, to establish which deaf children will benefit most from this program, and to clarify the mechanism by which the training is driving improvements in speechreading skills. However, the training program is likely to be of interest to any teachers of the deaf, speech and language therapists, or parents of deaf children who are looking for tools to improve a deaf child's speechreading skills.

The second hypothesis we sought to address was that the speechreading training program could support improvements in phonological and reading skills. One measure of phonological skill was a measure of speech production, which scored both auditory and visual (AV) components of the child's speech during picture naming of items, which were included in the speechreading training. Children in the speechreading group who had poorer AV speech output at the start of the study showed evidence of a significant benefit of speechreading training to their AV speech production, when compared to the control group. Although there were significant short-term benefits of the speechreading training to knowledge of the trained vocabulary, this would not account for the gains seen in the AV speech task, as scores on that task reflected the quality of phonological representations only of known vocabulary items. Instead, the results suggest that the children who completed the speechreading training were learning more about the phonology of words that they already knew (i.e., the specificity of their phonological representations was being improved).

While there was some evidence of benefits to phonological representations of the trained words from the AV speech production task, gains in phonological skills as measured by the phonological awareness task were small and nonsignificant. There was also no consistent evidence of an effect of the speechreading training on word reading. Although children improved in their reading proficiency over the course of the study, their reading performance, relative to others in the sample, was highly stable over time. This indicated little influence of the speechreading training on individual trajectories of reading development.

There are a number of potential reasons why we did not see larger downstream consequences of the speechreading training. First, the complete speechreading program only provided 8 hr of speechreading training over the course of approximately 3 months. This is in the context of the additional input that the children would have been receiving to foster their reading development during this time both at school and at home. Thus, the speechreading gains brought about by the speechreading intervention offered in this program may not have been of sufficient magnitude to bring about detectable gains in reading on their own. Although the current study included some elements of grapheme-to-viseme matching, this was not the primary focus of the training. It may be that speechreading training is most effective when fully embedded as part of a broader literacy program. Relatedly, the majority of this cohort of children had increased auditory access to the phonology of spoken language compared to previous generations of deaf children. Therefore, an intervention that capitalized on both available auditory and visual information about phonology, that is, involving AV speech stimuli, may have been more effective in helping to holistically develop their phonological representations (see, e.g., Bergeron, Lederberg, Easterbrooks, Miller, & Connor, 2009; Miller, Lederberg, & Easterbrooks, 2013; Summerfield, 1992). AV intervention was not used in the current study because our theoretical rationale focused on silent speechreading. In addition, deaf children are extremely variable in the auditory input they can receive from hearing aids and cochlear implants. Therefore, it was decided not to introduce this variability in the current study.

Aspects of the study design may also have limited its capacity to demonstrate benefits of the intervention. We selected children for this study based on age, rather than language level. Some children were already competent speechreaders and readers. It is likely that children with the poorest speechreading and reading skills are those who would benefit most from the training, though a minimum level of spoken language knowledge is also likely to be a prerequisite for benefit. The study may also have been limited in its power to detect effects of the intervention due to the relatively small sample size, compounded by issues around adherence to the intervention. The issue of small sample sizes in studies with special populations is a common limitation; more cross-center collaborative studies to increase participant numbers would be a valuable way to address this. Adherence to the intervention is also an issue that future studies should aim to address. Although conducted in schools, less than two thirds of children in each group completed the planned number of training sessions. Frequent issues encountered included consistency of support to help the child log on to the program and technical issues with school information technology systems.

Despite these issues, this study provides the first RCT to examine the efficacy of literacy interventions for deaf children. In their review of the literature on strategies for teaching deaf children grapheme–phoneme correspondences, Tucci, Trussell, and Easterbrooks (2014) highlighted the dearth of intervention studies in this area and argued that “the evidence base for literacy interventions in the field of deaf education is still in its infancy” (pp. 199). Deaf children deserve the same high-quality evidence base to inform their literacy instruction as hearing children, and increasing the size and quality of that evidence base should be a priority.

## Supplementary Material

10.1044/2019_JSLHR-H-19-0073SMS1 Supplemental Material S1 CONSORT 2010 checklist of information to include when reporting a randomised trial.

10.1044/2019_JSLHR-H-19-0073SMS2 Supplemental Material S2 Further details regarding training arms and assessments.
