# Supplementary material for: Computerized Speechreading Training for Deaf Children: A Randomized Controlled Trial
Source: J Speech Lang Hear Res. 2019 Jul 23;62(8):2882–94. doi: 10.1044/2019_JSLHR-H-19-0073 (PMC6839416; doi:10.1044/2019_JSLHR-H-19-0073)
Supplement: Supplemental Material S2 [file JSLHR-62-2882-s002.pdf]

Supplemental Material S2. Further details regarding training arms and assessments.

Title: Computerized Speechreading Training For Deaf Children: A Randomized Controlled Trial

Authors: Hannah Pimperton,[*++] Fiona Kyle,[+] Charles Hulme,[**] Margaret Harris,[***] Indie Beedie,[*++] Amelia Ralph-Lewis,[*++] Elizabeth Worster,[*] Rachel Rees,[+++] Chris Donlan,[+++] & Mairéad MacSweeney[*++]

[*]Institute of Cognitive Neuroscience, University College London, London, UK
[+]Language and Communication Science, City, University of London, London, UK
[**]Department of Education, University of Oxford, UK
[***]Faculty of Health and Life Sciences, Oxford Brookes University, Oxford, UK
[++]Deafness, Cognition and Language Research Centre, University College London, London, UK
[+++]Department of Language and Cognition, University College London, London, UK

Further Details Of Training Arms

Speechreading and maths 10 minute training sessions were made up of a combination of 4 different games with some narrative filler segments. All games were based on the theme of "space." At the beginning of each session the child was required to choose a space captain and a spaceship for their game that day. The first game involved "Packing a Rocket" (Figure 2a, main paper). There was then a "travel" in space to a planet game (Figure 2b). Once on the planet, a filler segment showed the captain meeting an alien. Children then played a game with the alien on the planet (Figure 2c, d, e, f). A different "planet" game was played each day. Once all 4 had been played, the first was played again. This provided some novelty to the training each day. After playing the "planet" game, a filler segment showed the alien giving the captain a prize. The captain then played a return "travel" game (Figure 2g). Once back on the captain's planet, the child was able to open the captain's prize and store this in their online trophy cabinet (Figure 1h).

<u>Speechreading Training</u>

Stimuli

103 words were included in the training dataset. All words were concrete nouns and were chosen because of their early age of acquisition. Art work was created for the games to represent the items (for examples, see Figure 2). These images were refined following tests of naming agreement with hearing 4-5yr old children. Four different talkers (three adults, one child; 2M,2F) were filmed saying each of the English spoken labels for the items aloud. Although the stimuli were recorded audio-visually, participants only ever saw visual-only videos of the spoken words. Children saw all four talkers saying all words throughout the course of the training to encourage them to learn to extract the commonalities between visual speech patterns of different talkers.

Game Design

At the beginning of each 10 minute speechreading training session the children completed a brief task that was designed to help them understand the relevance of good speechreading conditions in the real world. In these tasks the children had to get the models that they would see in the games ready so they could speechread them. For example, in one task they had to press a button that gradually turned up the light on one of the speechreading models until they could see their face well enough to speechread. In another, they had to press a button to make the model turn around until they were facing them.

The speechreading intervention comprised algorithm-based speechreading and reading training. The training was designed to run across 48 ten-minute sessions. The first 16 sessions contained trials that involved visual speech videos and pictures only. These focused on introducing

the vocabulary used in the intervention (103 words) and on mapping speechread words to a corresponding image. In these trials, children saw a silent video of a model saying the target word (e.g., "rabbit") and then saw a speech bubble overlaid on the video with the corresponding target image in it (i.e., they were given an explicit pairing of the visual speech token and a picture that the token referred to). They could then choose the correct target image from two response options. Immediately following this they would do a paired trial in which they would see the target image from the previous trial and had to choose the corresponding video with the target visual speech from a choice of two video response options (e.g., "rabbit" and "elephant"). For all trials, participants were free to articulate the perceived words if they chose to do so.

For the core speechreading trials, children saw a video of a model saying one of the 103 target words and then had to choose the corresponding picture from a choice of the target and three distractors. An algorithm was developed that enabled the difficulty level of these trials to be systematically varied in an adaptive way based on the child's performance. The adaptive algorithm was driven by varying the visual similarity between the target and the distractors based on the visual similarity of their constituent phonemes. To derive this visual similarity information, we collected data from British English-speaking hearing adults using an established paradigm (Auer & Bernstein, 1997) to determine the confusability of individual phonemes presented in the visual-only modality. Participant visual phonemic identification data produced confusion matrices (separately for vowel and consonant phonemes). Multidimensional scaling solutions were then applied to the confusion matrices to estimate visual phonetic similarity. To provide information for the speechreading algorithm on how visually similar any two words from the pool of 103 were, Similarity Choice Model similarity coefficients for each possible pair of phonemes were calculated. These allowed an estimate of the visual similarity between each of the 103 stimulus words and every other word based on the similarity of the constituent phonemes.

Creating the adaptive algorithm in this way meant that children would begin with targets and distractors that were highly visually distinct and would advance through to targets and distractors that were progressively more similar when they achieved criterion levels of success on easier trials. An example of progressively more difficult contrasts is: bee-fish > bee-boot > bee-bees> bee-pea. An example of an easy trial would be to match the spoken target "mat" to images of "mat, elephant, spoon, car," in which the overlap in visual speech between target and distractor pictures is very low. An example of a difficult trial would be to match "mat" to the target picture, selecting from "mat, map, hat, pan" in which the visual speech overlap is high.

In addition to trials operating at the single word level, children also completed trials which a) showed videos of two word utterances (e.g., "red hat"; "blue door") and b) showed videos of the two word utterances within a carrier sentence and hence required the child to identify the key information in the surrounding sentence (e.g., "find the red hat this time"). In both cases, these trials still involved video to picture matching.

Sessions 17 through 48 continued the speechreading training trials introduced in the first 16 sessions but additionally included trials that contained orthographic stimuli and that focused on training mappings between visual speech patterns and letters and words. These trials made up 50% of the trials played each day in sessions 17 to 48. The remaining 60% focusing on speechreading alone. These trials were designed to use visual speech to target the skills of grapheme-phoneme matching (e.g., seeing a video of a phoneme and choosing the corresponding letter or digraph), blending and segmenting (e.g., seeing a video of a word broken down into its constituent phonemes and choosing a picture that corresponded to the blended whole word), and spelling (e.g., seeing a video of a whole word and picking letters to spell that word).

The reading trials were rendered adaptive in two ways. First, the level of support was varied such that children moved through a systematic series of levels of difficulty on the same stimulus. For example, on easier blending trials the visual speech stimuli were accompanied by simultaneous corresponding orthographic stimuli. On more difficult trials the visual speech stimuli were presented with orthography and children had to derive the orthographic correspondence without support. On

easier spelling trials the words were broken down into their constituent phonemes and then blended to make the whole word. On more difficult trials the whole word was presented and the children had to segment the word themselves to complete the spelling task.

A second way in which the reading algorithm operated adaptively was by varying the complexity and regularity of the orthographic to phonological mapping of the words used. The words in the intervention were divided into six pools. Pool 1 contained words that were CVC in structure and contained regular orthography-phonology mappings involving a single letter to a single sound (e.g., "pig," "tap," "zip"). Pool 2 contained words that contained regular orthography-phonology mappings and included consonant digraphs in addition to single letter to single sound mappings (e.g., "chip," "fish," "king"). Pool 3 contained words that contained regular orthography-phonology mappings and included vowel digraphs in addition to single letter to single sound mappings (e.g., "coat," "moon," "tree"). Pool 4 contained words that had one or more complex or irregular orthography-phonology mappings (e.g., "ball," "knee," "wheel"). Pool 5 contained words that contained split digraphs (e.g., "bone," "cake," "kite"). Finally, pool 6 contained words that had complex mappings, were multisyllabic or did not fit in one of the previous pools (e.g., "elephant," "scissors," "trousers"). Reaching a pre-specified criterion level of success on each pool of words enabled the children to progress the subsequent pool.

Active Control Condition: Maths Training

The children in the control group played the same set of seven space-themed computer games as the children in the speechreading group, however the content of the games was number and maths trials not speechreading (see Figure 2 main paper for examples). Therefore, children in the two groups experienced the same visual environment and rewards, with the only difference being the skills being trained in the games. The maths content was driven by adaptive algorithms that presented early number skills, counting, and arithmetic trials that responded to the child's performance level.

Difficulty level was varied both by the numbers used (e.g., 1–10 vs. 10–20) and the operations required on those numbers. For example, moving from mapping objects to objects to mapping objects to digits; moving from completing sequences where numbers count up in 1 to sequences where numbers count up in 5; moving from completing additions where the sum remains on the screen to completing additions where the sum disappears and has to be retained and operated on in working memory.

Assessments

In-Game Assessments (IGAs)

There were seven IGAs in total, with the first assessment completed prior to the first session and final assessment at the end of the 48 training sessions. Therefore, only those who completed all of the training sessions, completed all of the IGAs. In each assessment trial the children viewed a video of talker saying one of the trained words and had to choose the corresponding picture from a choice of four. There were 30 trials in total, 15 of which used videos of the one of the talkers (n = 4) from the speechreading intervention (trained) and 15 parallel trials with the same target word and response options but which used videos of a model who was not included in the speechreading intervention (untrained). The same 30 trials were used for each of the IGAs. These IGAs were completed independently by the children during the training sessions and not administered by the researchers.

Assessments at Pre-Test (T1), Post-Test (T2), After Intervention Follow-Up at 3 Months (T3) and 11 Months (T4)

Pre-Specified Primary Outcome Measure: Test of Child Speechreading (ToCS)–Core Test

The ToCS core test (Kyle et al., 2013) starts with a familiarization task in which children see a silent video of the two models who produce the test stimuli saying the days of the week. Each of the three subtests follows a similar format, beginning with practice trials in which explicit feedback is given, followed by test trials in which no feedback is given. The children watch a silent video of a model saying a word, sentence or short story and then must choose a picture which corresponds to their answer from a choice of four. For the words and sentences subtests, the picture chosen must correspond to what the model said. For the short stories part of the assessment, the tester asks the child two questions about each story and they must choose a picture that answers the question asked. There are 15 trials in the words and sentences subtests and 10 in the short stories subtest, giving each child a total raw score out of a possible 40.

Pre-Specified Secondary Outcome Measures: Speechreading: Test of Child Speechreading (ToCS) – Everyday Questions Test

Children were required to watch silent videos (*n* = 12) of two talkers asking questions they might encounter in everyday life (e.g., where do you live?) and tell the experimenter what they thought the question was (Kyle et al., 2013). Children could answer using their preferred communication mode. Children received two scores on this measure, one reflecting the number of questions they correctly reproduced the gist of (ToCS Everyday Questions Items Correct Gist), and one reflecting the total number of individual words that the child got correct across all 12 questions out of a possible 62 (ToCS Everyday Questions Words Identified). For example, if the question was "how old are you?" and the child's response was "how are you?" they would receive 0 on that item for the Items Correct Gist score but 3 for the Words Identified score. If the question was "what did you eat for breakfast?" and the child's response was "what did you have for breakfast?" they would receive 1 on that item for the Items Correct Gist score and five (out of a possible six) for the Words Identified score. The responses were transcribed online during the testing session, checked and scored offline from the video by the tester, and then checked from the video by a second blinded scorer.

Vocabulary

A naming task, using the pictures from the training, was used to assess participants' knowledge of the vocabulary used in the speechreading training. Their first response was taken for each trial. If they named it in sign, they were asked if they knew the English word. Each participant was given a score for the number of correct items produced in spoken English (Spoken Vocabulary; total = 74) and a score for the number of correct items produced either in spoken English or BSL, thus providing a measure of overall vocabulary, regardless of modality (Overall Vocabulary; total = 74).

Audio-Visual Speech Production (AV Speech Production)

Participants were filmed completing the picture naming task described above. For the purposes of obtaining a speech production score, if the child named the picture incorrectly or could not name it at all on their first attempt, the experimenter provided them with the correct label and asked them to repeat it.

The 30 words selected for this measure were chosen to maximize the range of phonemes in syllable-initial and syllable-final positions, and to provide a range of word lengths and syllable structures, including consonant clusters. To calculate a score that reflected changes in the quality of phonological representations of the same words over time for each child, items that were named incorrectly or not attempted at any of the time points were excluded from the analysis. Attempts

that were phonologically unrelated to the target word were also excluded, to avoid random vocalizations. Thus, each child received a total possible score based on the words that they attempted at all time-points. This was used to obtain their overall score at each time point as a percentage of the possible score. Thus this score can be interpreted as a measure of the extent to which the child updated their phonological representation of words attempted at each time point.

A narrow transcription was made for each word, based on the International Phonetic Alphabet (IPA). Each consonant was then scored according to the following scoring system: Correct within the boundaries of the target phoneme or an acceptable allophone, including accent variations (4 points); Place correct plus either voice or manner correct (3 points); Place correct but voice and manner are incorrect or for all target consonants further back than dental, place not correct but within the wider category (i.e., coronals, velars, glottals) or silent articulations in the correct place or place in the wider category or clicks in the correct place or place in the wider category (2 points); place incorrect or (for target consonants further back than dental), not within the wider category (1 point); omission (0 points). The maximum score for each consonant was 4, and each word had a maximum score based on the number of consonants. The maximum total for all 30 words was 284. To verify the reliability of transcriptions, a second marker transcribed and scored a subset of 10% (6 children/540 words/1278 consonants) of the data. Agreement between the two scorers was good (Cohen's Kappa = 0.71, *SE* = 0.02).

Phonological Awareness

All stimuli in the phonological awareness tasks were stimuli included in the speechreading training. In the onset trials (*n* = 12), children viewed a target picture (e.g., house) and had to choose the item from a choice of three (e.g., hand; cow; jam) that started with the same sound. One of the incorrect distractors overlapped with the target in terms of vowel (near distractor; e.g., cow). The rime trials (*n* = 12) followed the same format. In this case, the correct response (e.g., peg) shared the rime with the target (e.g., leg). The near distractor shared the vowel with the target (e.g., bell).

Letter-Sound Knowledge

The letter-sound productions were scored online during the testing session, checked offline from the video by the tester, and subsequently checked offline from the video by a second blinded scorer. This assessment was not carried out at T4.

Word Reading

Three measures were used to assess the children's word reading ability. The first two were taken from the YARC (Snowling et al., 2009) and assessed single word reading of untrained stimuli. The early word recognition test (EWRT) is designed for 4–7 year olds and assesses children's ability to read 30 early acquired words. The single word reading test (SWRT) was also administered to avoid any ceiling effects as it was designed for 5–11 year olds and hence contained more challenging words (*n* = 60). Children who used BSL as their preferred communication mode labeled the word in sign rather than reading it aloud in English. These reading measures were scored online during the testing session, checked offline from the video by the tester, and subsequently checked offline from the video by a second blinded scorer.

The third reading measure was a novel test that assessed single word reading for stimuli included in the speechreading training (*n* = 24 trials). Children saw a word in the middle of the screen and had to point to the corresponding picture from a choice of four, therefore no speech production was required. A reading composite score was created by summing each child's *z* scores on the three word reading measures.

Number Skills

Three measures of number skills were administered. (1) The Early Number Concepts section of the BAS-III (Elliot & Smith, 2011) provided a measure of children's understanding of concepts

related to number (e.g., “more than,” “less than”) and early number skills (e.g., counting, adding, subtracting). (2) A standardized measure of addition and subtraction fluency taken from the Test of Basic Arithmetic and Numeracy Skills (Hulme, Brigstocke, & Moll, 2016). (3) Children were asked to count to 30, with the highest number they could reach being their score on this task. A Number Skills composite score was created by summing each child’s *z* scores on the three measures of number skills. At T4 only the measure of addition and subtraction fluency was administered.

References

Auer, E. T., Jr., & Bernstein, L. E. (1997). Speechreading and the structure of the lexicon: Computationally modeling the effects of reduced phonetic distinctiveness on lexical uniqueness. *The Journal of the Acoustical Society of America, 102*(6), 3704–3710.

Elliot, C. D., & Smith, P. (2011). British Ability Scales–Third Edition. London, United Kingdom: GL Assessment.

Kyle, F. E., Campbell, R., Mohammed, T., Coleman, M., & MacSweeney, M. (2013). Speechreading development in deaf and hearing children: Introducing the Test of Child Speechreading. *Journal of Speech, Language, and Hearing Research*, *56*(2), 416–426.

Hulme, C., Brigstocke, S., & Moll, K. (2016). *Test of Basic Arithmetic and Numeracy Skills*. Oxford, United Kingdom: Oxford University Press.

Snowling, M. J., Stothard, S. E., Clarke, P., Bowyer-Crane, C., Harrington, A., Truelove, E., . . . Hulme, C. (2009). *York Assessment of Reading for Comprehension.* London, United Kingdom: GL Assessment.
